# Stiffening of graphene oxide films by soft porous sheets

**DOI:** 10.1038/s41467-019-11609-8

**Published:** 2019-08-15

**Authors:** Lily Mao, Hun Park, Rafael A. Soler-Crespo, Horacio D. Espinosa, Tae Hee Han, SonBinh T. Nguyen, Jiaxing Huang

**Affiliations:** 1 Department of Chemistry, Northwestern University, 2145 Sheridan Rd., Evanston, IL 60208 USA; 2 Department of Organic and Nano Engineering, Hanyang University, Seoul, 04763 Republic of Korea; 3 Department of Materials Science and Engineering, Northwestern University, 2220 Campus Dr., Evanston, IL 60208 USA; 4 Theoretical and Applied Mechanics Program, Northwestern University, 2145 Sheridan Rd., Evanston, IL 60208 USA; 5 Department of Mechanical Engineering, Northwestern University, 2145 Sheridan Rd., Evanston, IL 60208 USA

**Keywords:** Characterization and analytical techniques, Two-dimensional materials, Structural properties

## Abstract

Graphene oxide (GO) sheets have been used as a model system to study how the mechanical properties of two-dimensional building blocks scale to their bulk form, such as paper-like, lamellar-structured thin films. Here, we report that the modulus of multilayer GO films can be significantly enhanced if some of the sheets are drastically weakened by introducing in-plane porosity. Nanometer-sized pores are introduced in GO sheets by chemical etching. Membrane-deflection measurements at the single-layer level show that the sheets are drastically weakened as the in-plane porosity increases. However, the mechanical properties of the corresponding multilayer films are much less sensitive to porosity. Surprisingly, the co-assembly of pristine and etched GO sheets yields even stiffer films than those made from pristine sheets alone. This is attributed to the more compliant nature of the soft porous sheets, which act as a binder to improve interlayer packing and load transfer in the multilayer films.

## Abstract (web summary)

Graphene oxide sheets have been used as a model system to study how the mechanical properties of individual 2D building blocks scale to their bulk form. Here the authors show that the modulus of multilayer graphene oxide films can be enhanced if some of the sheets are weakened by introducing in-plane porosity.

## Introduction

The large lateral dimension of high-aspect-ratio two-dimensional (2D) materials facilitates property measurements at the single-layer level^1–4^. They can also easily assemble into bulk continuous solids^5^. Therefore, 2D sheets provide a unique opportunity to study how nanoscale properties scale to their bulk forms. A well-studied example is graphene oxide (GO), an oxygenated derivative of graphene sheets that can easily disperse in water^6^. Their excellent solution processability, uniform thickness, and high-aspect ratio make GO sheets a good model system to study how material properties scale from single-layer building blocks to their bulk structures. When filtered or casted from solution, GO sheets readily assemble into macroscopic films with a lamellar microstructure^7–9^. There is extensive interest in understanding how the overall mechanical properties of such multilayer lamellar films are affected by the nanoscale structure of the constituent sheets and the interlayer interactions between sheets^7,10^. Herein, we discover that the modulus of multilayer GO films can be significantly enhanced if some of the sheets are drastically weakened by introducing in-plane porosity. At the single-layer level, the elastic modulus of GO sheets decreases rapidly as their porosity increases, but becomes much less sensitive for their corresponding multilayer films. Surprisingly, co-assembly of pristine GO sheets and the much-weaker, high-porosity sheets leads to even stiffer GO films. These results help to reveal a dilemma in interlayer stacking, which prevents the mechanical properties of pristine, un-etched GO sheets to scale up in multilayer films. Since porous GO sheets are much softer and compliant, they can effectively act as a binder to improve interlayer interaction and packing, leading to GO films with much higher modulus.

## Results

### Synthesis of porous GO

**Fig. 1:**
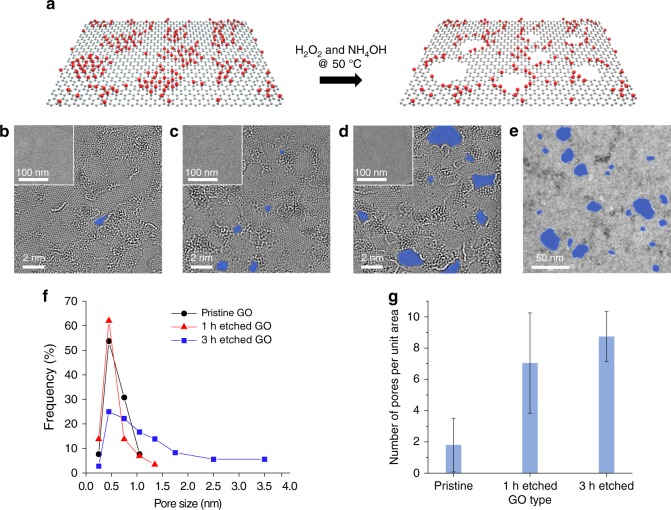
Controlled oxidative etching of GO yields single layers with tunable porosity. **a** Schematic models of GO and porous GO. Red dots represent sites with oxygen-containing functional groups, which are preferentially etched, leaving holes on the graphene sheet. HR-TEM images of GO sheets **b** before and after being etched for **c** 1 h, and **d** 3 h, respectively. Pores are highlighted in blue color. The corresponding lower-magnification TEM images are shown in the insets. **e** A low-magnification TEM image of the 5-h-etched GO sample, showing extensive formation of large pores. **f** Size distribution and **g** number density of pores found on the starting and etched GO sheets, based on a survey over an area of 500 nm^2^ in HR-TEM images. Error bars represent the standard deviation (SD)

GO sheets were synthesized by a modified Hummers method^11,12^ and purified by a two-step washing procedure to remove ionic contaminations^13^, which have been found to have a significant impact on the mechanical properties of GO films^14^. Porous GO sheets were made by oxidative etching using a mixture of ammonia solution and hydrogen peroxide (Fig. 1a). Etching starts preferentially at the less-stable oxidized *sp*^3^ domains in GO sheets^15,16^, and the reaction time can be varied to tune porosity without significantly altering the average sheet size (Supplementary Methods and Supplementary Fig. 1). The nanopores on GO sheets can be visualized by high-resolution transmission electron microscopy (HR-TEM) (Fig. 1b–e). A survey over an area of 500 nm^2^ of the TEM images confirmed that both pore size (Fig. 1f) and the number of pores (Fig. 1g) increased as the etching time was extended. After etching, the degree of oxidation is also slightly decreased for the porous sheets based on X-ray photoelectron spectroscopy (XPS) studies (Supplementary Note 1 and Supplementary Fig. 2). The conductivities of the starting GO and porous GO papers are in the range of 10^−5^–10^−4^ S cm^−1^, which is 4–5 orders of magnitude lower than the conductivity of typical reduced GO samples (e.g., 1–10 S cm^−1^). This suggests that under the etching conditions, GO sheets have not been significantly reduced. The main reason for the small change in C/O ratios observed through XPS can be attributed to selective removal of sp^3^-rich domains.

### Nanomechanical properties of single-layer porous GO

**Fig. 2:**
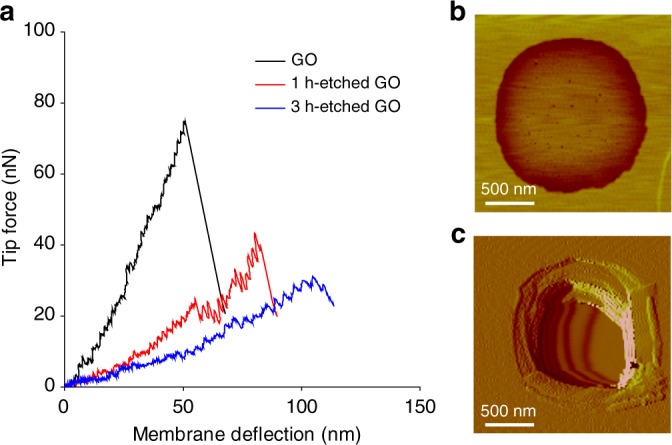
Nanomechanical characterization for pristine and porous GO single layers. **a** Representative force-deflection curves obtained by membrane-deflection experiments using a diamond AFM probe, suggesting etched sheets become softer and weaker as etching time increases. The 5-h-etched GO sheets are too weak to be measured. Representative AFM images of 3-h-etched GO prior to deflection experiments show visible pores in **b**, and complete rupture of the membrane after the test in **c**

Pristine and etched GO single-layer sheets were suspended via Langmuir-Blodgett deposition^4,12^ over an array of circular microwells pre-fabricated on a silicon substrate (Supplementary Methods). The center of the membranes was deflected with a single-crystal diamond probe equipped on an atomic force microscope (AFM), to determine mechanical properties (Supplementary Methods). Figure 2a shows typical force-deflection responses obtained for pristine, 1-h-etched, and 3-h-etched GO sheets. In all cases, an abrupt increase in deflection occurs as force drops due to film rupture. Representative AFM images of 3-h-etched GO show visible pores prior to deflection experiments (Fig. 2b), and complete membrane rupture after indentation (Fig. 2c). Notably, the tapping force imparted on the 3-h-etched GO membranes during AFM topography scans had to be significantly reduced to prevent membrane rupture before deflection experiments. And the 5-h-etched GO was found to be too weak to even suspend over the microwells for measurement (Supplementary Methods).

The elastic modulus of pristine GO membranes (*E* = 282.8 ± 20.6 GPa), obtained from an analysis of the force-deflection curves (Supplementary Fig. 3), is in good agreement with previous reports in the literature^2,4^. This value drastically decreases to 85.0 ± 12.0 GPa and 36.0 ± 10.9 GPa for 1 h and 3-h-etched GO, respectively (Supplementary Table 1 and Supplementary Fig. 4), suggesting a major impact due to porosity. While the stiffness and rupture force of these porous GO samples also substantially decreases, the deflection at rupture increases, suggesting increased ductility (Fig. 2a). We attribute this dichotomy to the presence of the nanopores in the etched GO sheets, which can effectively inhibit crack propagation and delay membrane rupture.

### Mechanical properties of pristine and etched GO multilayer films

**Fig. 3:**
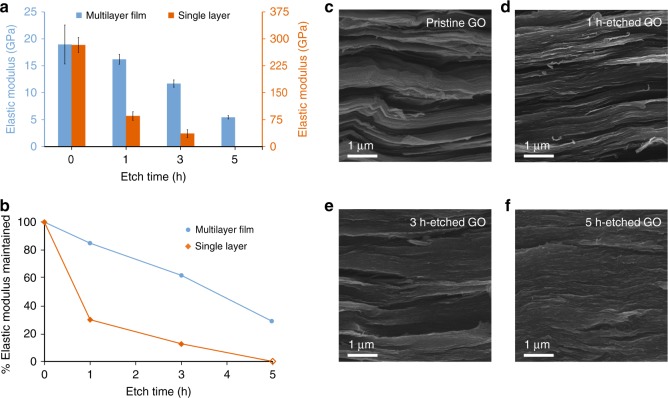
Scaling of mechanical properties from single layers to multilayer films. **a** Elastic moduli of single-layer pristine and etched GO sheets and the corresponding multilayer films. Error bars represent SD. **b** Percentage reduction of the elastic moduli of GO sheets as etching time increases, showing that multilayer films are much more tolerant to etching than single layers. SEM fractographs (i.e., images of the fracture surfaces) of multilayer films of **c** pristine, **d** 1-h-etched, **e** 3-h-etched, and **f** 5-h-etched GO sheets

Pristine and etched GO sheets were assembled by filtration to form paper-like multilayer lamellar films with thickness in the range of 7–11 µm. Uniaxial tensile tests were performed to examine the effect of porosity on their mechanical properties (See Supplementary Methods for experimental details). While porosity greatly affects the stiffness and strength of GO sheets at the single-layer level (Fig. 3a), its effects on the lamellar films are not as pronounced (Supplementary Table 2 and Supplementary Fig 5a, c, e). For example, while 1-h-etched GO single layers are only 30% as stiff as pristine sheets, the corresponding films are actually 87% as stiff as films made of pristine, un-etched GO. Films comprising 3-h-etched and 5-h-etched GO sheets similarly exhibited a much higher relative stiffness with regard to pristine, un-etched GO (62% and 28%, respectively) than their single-layer constituents (13% and ~0%, respectively) (Fig. 3b). For single-layer GO sheets, the membrane-deflection measurements directly probe their intrinsic mechanical properties. However, for multilayer films, their tensile properties are determined by both the properties of the single-layer building blocks, as well as the interlayer load transfer. While the presence of nanopores greatly weakens the single layers, it does not significantly reduce the overlapping area, hence load transfer between the layers in the lamellar films. The striking difference in how porosity affects the mechanical properties of GO at the single-layer vs. multilayer level confirms that interlayer load transfer dominates the tensile properties of the lamellar films^2,10,17^.

After tensile tests, the fracture surface of the multilayer films was imaged by scanning electron microscopy (SEM), revealing denser packing of constituent sheets with increased etching time (Fig. 3c–f). While pristine GO films exhibit uneven fracture surfaces with voids (Fig. 3c), 5-h-etched GO films exhibit very smooth and uniform fracture surfaces (Fig. 3f), suggesting that highly porous sheets can pack together more efficiently. These SEM observation suggests that although the porous GO sheets become much weaker and softer at the single-layer level, they can pack more uniformly and tighter, thus allowing more effective interlayer load transfer to offset porosity-induced decrease in stiffness.

### Mechanical properties of mixed pristine and etched GO multilayer films

**Fig. 4:**
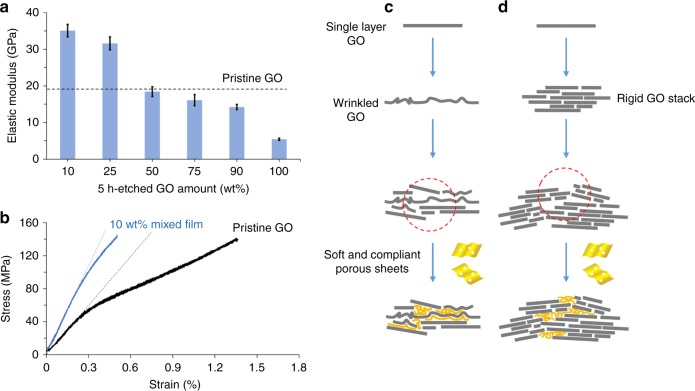
Stiffening of GO films by soft porous sheets. **a** Elastic moduli of mixed multilayer films with various fractions of 5-h-etched GO. Error bars represent SD. **b** Representative stress–strain curves of a pristine GO film and another with 10 wt% of 5-h-etched GO. Dashed lines represent the calculated elastic moduli of the films. These results revealed an intrinsic dilemma for pristine GO sheets that prevents them from continuously conformal stacking. **c** First, GO sheets are easily wrinkled or folded during processing, which disrupts the packing of neighboring sheets. **d** Second, even if wrinkle-free sheets tightly stack, they form platelets that are drastically more rigid, and less compliant, preventing for further conformal packing. Both **c** and **d** result in architectural defects (red circles) in the multilayer films, which would become stress concentrators to degrade the overall mechanical properties. Thus, the much softer porous GO sheets effectively serve as a binder, allowing more compliant packing in the multilayer films, leading to higher stiffness

Inspired by the aforementioned results, we hypothesized that the porous sheets may also improve the interlayer load-transfer of the pristine, un-etched GO sheets. Thus, 5-h-etched and pristine GO sheets were co-assembled to make multilayer films to test this hypothesis (Fig. 4 and Supplementary Fig. 5b, d, f). A series of GO papers containing 10, 25, 50, 75, and 90 wt% of 5-h-etched GO sheets were fabricated and subjected to uniaxial tensile testing (Supplementary Methods). Mixed films containing 90 wt% of 5-h-etched GO sheets maintain 62% of the strength of a pristine GO film, whereas pure 5-h-etched GO films retain only 30% (Supplementary Table 2, cf. entries 9 and 4). Furthermore, the tensile strength of the 10 wt% mixed film remained similar to that of a pristine GO film (Supplementary Table 2, cf. entry 5), confirming that the detrimental effects of porosity can indeed be mitigated by co-assembling etched and pristine sheets. Figure 4a compares the elastic modulus of films made of mixed sheets, with regard to that of a pristine GO paper. Surprisingly, the 75 and 90 wt% mixed films were nearly as stiff as a pristine GO film (Supplementary Table 2, cf. entries 8 and 9 vs. 1), despite the majority of the sheets being highly porous. The 25 wt% and 10 wt% mixed films exhibited a 1.7 and nearly twofold increase, respectively, in elastic modulus over that of the pristine GO film (Supplementary Table 2, cf. entries 5 and 6 vs. 1). It is quite remarkable that the modulus of GO films can actually be significantly enhanced when a small fraction of the constituting sheets become weaker.

Together, the results shown in Figs. 3 and 4a imply that optimized lamellar packing cannot be achieved with pristine GO sheets alone. Although this may seem counter-intuitive, it can be explained by the following dilemma in the stacking of 2D sheets. GO papers comprise sheets with very large aspect ratios, which are easily wrinkled and folded under external stress^18^. As illustrated in Fig. 4c, these uneven features will disrupt the interlayer packing of neighboring sheets and become weak interlayer links, reducing load transfer. On the other hand, if wrinkle-free sheets indeed pack conformally to form multilayers, it will generate thick slabs with drastically increased stiffness, which would not pack conformally and densely in the final lamellar structure (Fig. 4d). Therefore, 2D sheets alone are unlikely to generate lamellar films without interlayer voids. And such architectural defects can be compensated by introducing the much more compliant, porous GO as a filler and binder to improve interlayer load transfer. Figure 4b shows the stress–strain curves of a pristine GO film and a 10 wt% mixed film. For the pristine GO film, its stiffness decreases with increasing strain as GO sheets slide past each other and the film experiences plastic deformation^7^. In contrast, the stiffness of a 10 wt% mixed film remains similar up to film fracture, suggesting that adding porous sheets indeed has improved binding between layers, making the film more resistant to tensile stress.

### Lap-shear tests and fractographic study of delaminated surfaces

**Fig. 5:**
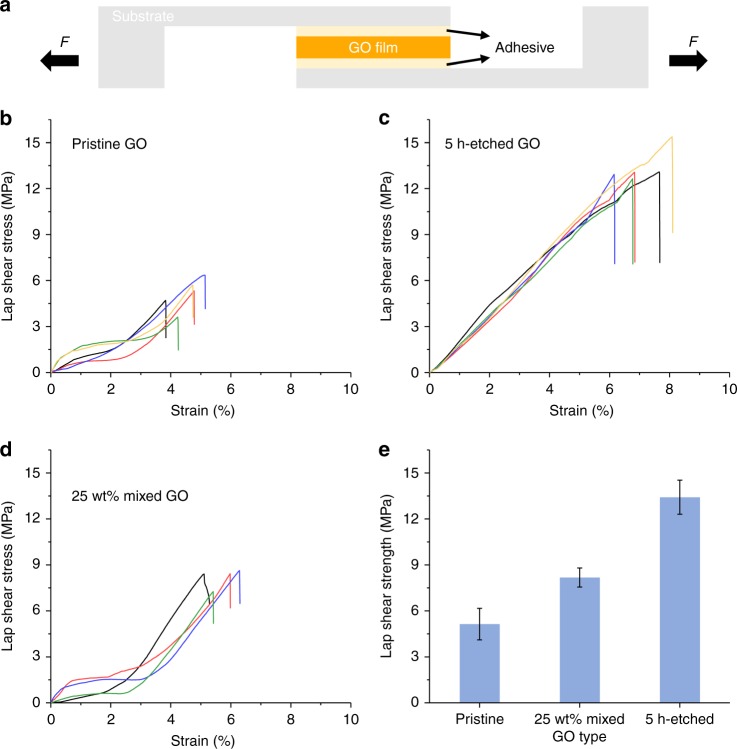
Lap-shear tests of GO films. **a** Schematic drawing showing the experimental configuration of the lap-shear test. A rectangular GO film (3 × 4 mm, around 10 µm thick) was glued between two parallel glass substrates using an epoxy adhesive. A shear stress was then applied by pulling the substrates in the opposite direction at a speed of 0.3 mm min^−1^ up to film delamination. Shear stress–strain curves of films made of **b** pristine, un-etched GO, **c** 5-h-etched porous GO, and **d** pristine GO mixed with 25 wt% of 5-h-etched GO. For each type of GO film, 4–5 samples were tested. For all the samples shown here, delamination occurred from within the GO film, rather than at the adhesive-GO or adhesive-glass interfaces. **e** Summary of shear strength values for all measurements in **b**–**d**. Error bars represent SD

The interlayer binding strengths of three types of GO films made from un-etched, pristine sheets, neat 5-h-etched sheets, and pristine sheets mixed with a 25 wt% of 5-h-etched sheets, respectively were studied using the lap-shear test configuration shown in Fig. 5a. In each test, a rectangular GO film (3 × 4 mm) was fixed in between two parallel rigid glass slides using an epoxy adhesive. Next, shear stress was applied by pulling the slides in the opposite direction at a rate of 0.3 mm min^−1^. In all cases, the films were delaminated from within the GO film rather than from the GO/adhesive or adhesive/glass interface, so that the measurement reflects the interlayer binding strength within the GO film. Figure 5b–d shows the lap-shear stress–strain curves of the three GO films, with 4–5 tests each. The values of the lap-shear stress at delamination are summarized in Fig. 5e. Films made of 5-h-etched sheets are found to have the highest shear strength (13.4 ± 1.1 MPa) and exhibit relatively simple elastic behaviors before delamination without any obvious plastic deformation (Fig. 5c). This is consistent with a uniform propagation of the shear deformation throughout the film, indicative of a tight interlayer binding between the porous sheets. In contrast, films made of pristine, un-etched GO sheets have the lowest shear strength (5.1 ± 1.0 MPa). Their shear stress–strain curves (Fig. 5b) show evidence of initial flow before reaching a deformation state similar to the elastic state observed in the 5-h-etched sample. This behavior implies that the pristine GO sheets experience partial interlayer sliding and rearrangement at the initial stage of shearing that eventually leads to a more “tightly bound” state. The mobility of the GO sheets during this stage of rearrangement could be facilitated by the presence of the architectural defects illustrated in Fig. 4c, d. Consistent with these observations, the films made from pristine GO mixed with 25 wt% of 5-h-etched GO were found to have an intermediate shear strength 8.2 ± 0.6 MPa, and a plastic-to-elastic transition in their shear stress–strain curves (Fig. 5d), similar to those of pristine, un-etched GO.

**Fig. 6:**
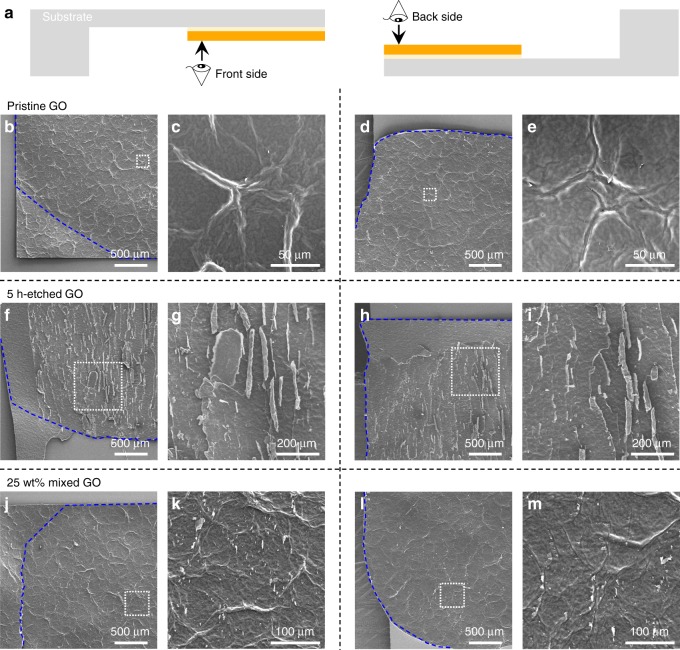
SEM fractographic study after delamination. **a** Schematic drawing illustrating a GO film delaminated after lap-shear test, which generate two fractured surfaces denoted as front and back sides. For the films made from pristine, un-etched GO sheets, SEM images reveal **b**, **c** cellular patterns of “upward” wrinkles on one side of the delaminated surface, and **d**, **e** complementary “downward” wrinkles on the other side. For the films made from 5-h-etched porous GO sheets, **f**–**i** both sides have terrace-like structure with some flakes being nearly pulled out. For the films made from a mixture of pristine and 25 wt% of 5-h-etched porous GO sheets, **j**–**m** the fractured surfaces show both wrinkles and terraces. The blue dashed lines in the low-magnification images (**b**, **d**, **f**, **h**, **j**, **l**) indicate the area of epoxy adhesive behind the delaminated GO. The higher-magnification images (**c**, **e**, **g**, **i**, **k**, **m**) are taken in the areas indicated by the white boxes in the corresponding low-magnification images

To better understand the delamination behaviors of the film, we image the front and back sides of the delaminated GO surfaces (Fig. 6a) using SEM. Figure 6b–e shows the morphology at the front and back sides, respectively, of a delaminated film made from un-etched, pristine GO sheets. Both sides have cellular patterns of wrinkles with cells of hundreds of microns in size. The wrinkles on the two sides are topologically complementary to each other, which is indicative of poor shear load transfer within the lamellar GO paper. In contrast, the delaminated surfaces of the films made from 5-h-etched GO sheets exhibit terrace-like fracture structures (Fig. 6f, g, front sides and Fig. 6h, i, back sides), suggesting a crack propagation path across the layers as a result of strong interlayer load transfer. Figure 6j–m shows the SEM images of the front and back sides of a delaminated film made from pristine GO mixed with 25 wt% of 5-h-etched GO sheets, respectively. These films exhibit an intermediate morphology, showing both wrinkles and terraces in-between the wrinkles, which can be attributed to the presence of porous sheets that act as a compliant filler and binder to enhance the interlayer load transfer of pristine, un-etched GO sheets.

## Discussion

GO sheets have been used as a model system to construct bulk form of materials from 2D building blocks. The study here suggests that 2D sheets alone cannot be used to construct bulk lamellar structured papers with effective interlayer load transfer, due to the dilemma in sheet-stacking. To this end, the modulus of multilayer GO films can be significantly enhanced by adding some porous sheets, which are drastically weaker than pristine, un-etched ones but can enhance the interlayer load transfer of the former. The work here also shows the drastically different effect of in-plane porosity on the mechanical properties of single layers and multilayer papers. These insights should be largely materials agnostics, and may be applicable to other bulk form of materials made from 2D nanomaterials.

## Methods

### Synthesis of porous GO

Single-layer GO was prepared using a modified Hummers method^11,12^, and porous GO was produced by etching the as-synthesized GO with a mixture of ammonia solution and hydrogen peroxide (See Supplementary Methods for experimental details).

### Mechanical tests of single- and multi-layer samples

For AFM membrane-deflection experiments, suspended single layers of pristine (un-etched) and etched GO were prepared via Langmuir–Blodgett deposition onto patterned Si substrates^4,12^. Multilayer lamellar films of pristine and etched GO were prepared through vacuum-assisted filtration of the corresponding aqueous dispersions. Mixed films were similarly obtained by vacuum filtration of premixed aqueous dispersions of pristine and 5-h-etched GO. The mechanical properties of the multilayer films were measured by uniaxial tensile testing and lap-shear tests. For methodological details, see Supplementary Methods.

## Supplementary information

Supplementary Information

## Data Availability

The data that support the findings of this study are available from the corresponding author upon reasonable request.
