# Supplementary material for: Stiffening of graphene oxide films by soft porous sheets
Source: Nat Commun. 2019 Aug 15;10:3677. doi: 10.1038/s41467-019-11609-8 (PMC6695419; doi:10.1038/s41467-019-11609-8)
Supplement: Supplementary file 1 — Supplementary Information [file 41467_2019_11609_MOESM1_ESM.pdf]

Supplementary Information

Stiffening of Graphene Oxide Films by Soft Porous Sheets

Lily Mao[1,6], Hun Park[2,3,6], Rafael A. Soler-Crespo[4,6] Horacio D. Espinosa[4, 5], Tae Hee Han[2*], SonBinh T. Nguyen[1*], Jiaxing Huang[3*]*

[1]Department of Chemistry, Northwestern University, 2145 Sheridan Rd., Evanston, IL 60208, USA.
[2]Department of Organic and Nano Engineering, Hanyang University, Seoul 04763, Republic of Korea.
[3]Department of Materials Science and Engineering, Northwestern University, 2220 Campus Dr., Evanston, IL 60208, USA
[4]Theoretical and Applied Mechanics Program, Northwestern University, 2145 Sheridan Rd., Evanston, IL 60208, USA.
[5]Department of Mechanical Engineering, Northwestern University, 2145 Sheridan Rd., Evanston, IL 60208, USA.

[6]These authors contributed equally.

[*]Corresponding authors: espinosa@northwestern.edu (H. D. Espinosa), than@hanyang.ac.kr (T. H. Han), stn@northwestern.edu (S. T. Nguyen), jiaxing-huang@northwestern.edu (J. Huang)

Table of Contents

Supplementary Figure 1. SEM analysis of pristine and etched GO sheet thickness and size
Supplementary Figure 2. XPS and Raman spectra of GO and porous GO samples
Supplementary Figure 3. Fitting procedure for linear-elastic, membrane-deflection model
Supplementary Figure 4. Summary statistics for elastic modulus and pre-stress in pristine and etched GO
Supplementary Figure 5. Summary statistics for mechanical properties of multilayer GO films

Supplementary Table 1. Elastic modulus and pre-stress of single-layer GO sheets.
Supplementary Table 2. Elastic modulus and tensile strength of multilayer films.

Supplementary Methods: Materials and instrumentation, Synthesis of GO and porous GO, Preparation of Si substrates with microwells, LB assembly, AFM membrane-deflection tests, Fabrication and mechanical analysis of multilayer films

Supplementary Note 1. XPS and Raman characterization of pristine and etched GO sheets
Supplementary Note 2. Analysis of membrane-deflection tests

Supplementary References

Supplementary Figures

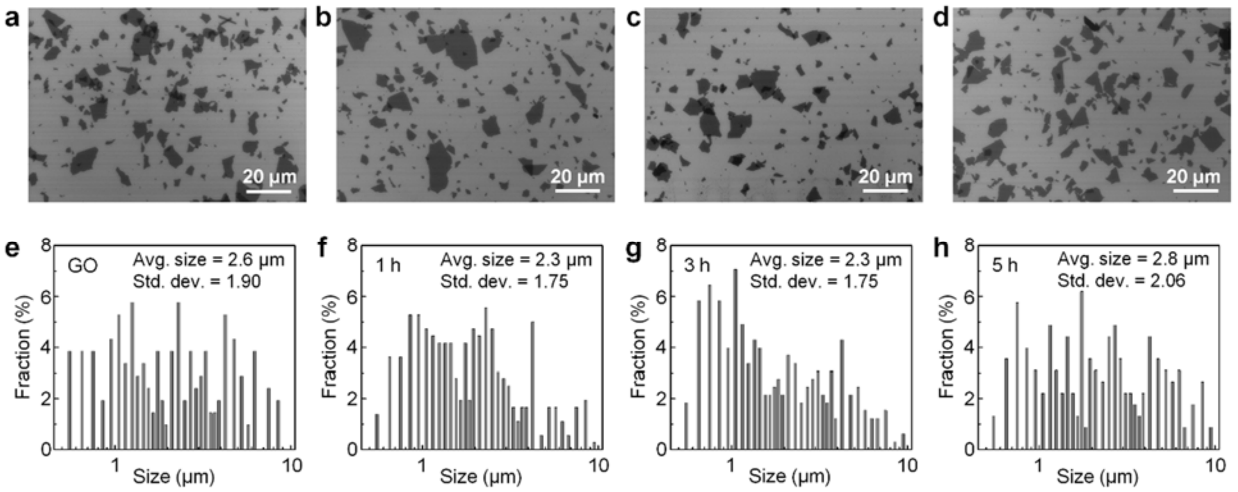

Supplementary Figure 1. SEM analysis of pristine and etched GO sheet thickness and size. (a-d) Representative SEM images of: (a) pristine, (b) 1 h-etched, (c) 3 h-etched, and (d) 5 h-etched GO sheets. (e-h) Corresponding size distributions of (e) pristine, (f) 1 h-etched, (g) 3 h-etched, and (h) 5 h-etched GO sheets, obtained from SEM image analysis. The average sheet size does not change significantly with etching time, suggesting that the nanopore etching process does not initiate at the sheet edges.

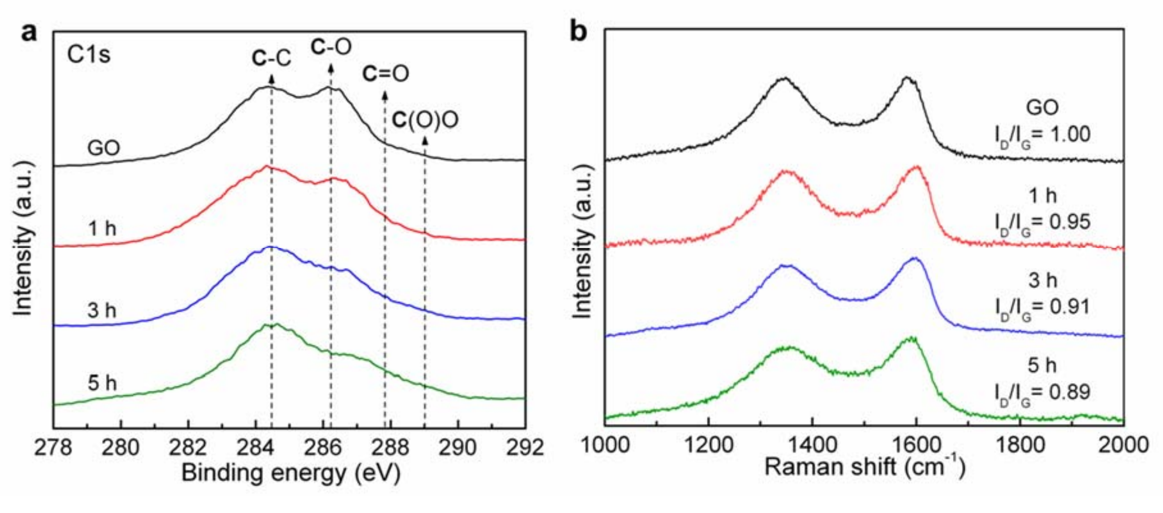

Supplementary Figure 2. XPS and Raman spectra of GO and porous GO samples. (a) C1s XPS scans of pristine and etched GO, showing a decrease in oxidized carbons with increasing etching time. (b) Raman spectra of pristine and etched GO. The I_D/I_G ratio decreases with etching time.

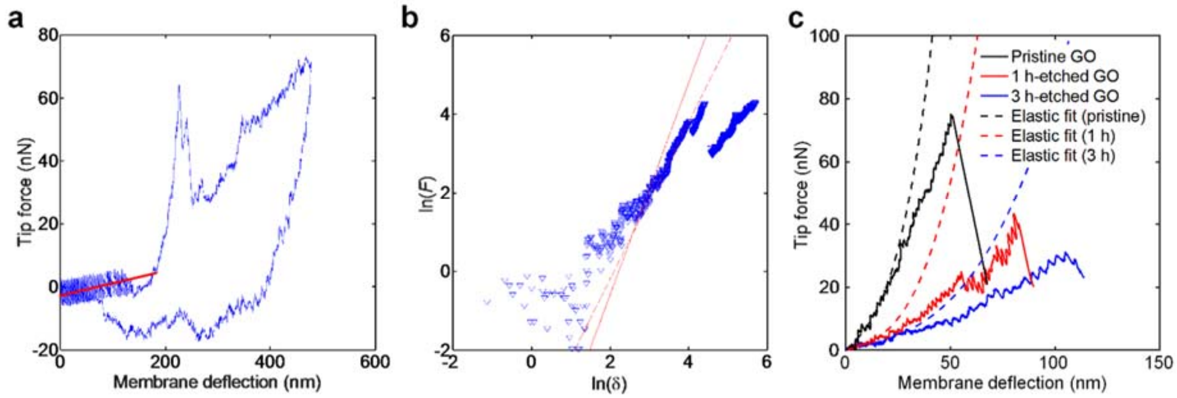

Supplementary Figure 3. Fitting procedure for linear-elastic, membrane-deflection model. (a) Selection of the first point for fitting force-deflection curves. This point corresponds to the deflection when the tip effective force, post-membrane adhesion (i.e., after the cantilever straightens after snapping into the membrane), matches the average force measured during tip approach. (b) Selection of the last point for fitting force-deflection curves, when the experimental data deviates from the slope (i.e., slope of 3 in the ln δ term) given by Supplementary Equation 2. The left fit corresponds to a slope of 3, while the right fit corresponds to a slope of 2. (c) Representative data fit for the force-deflection curves shown in Fig. 2a, using the criteria outlined herein.

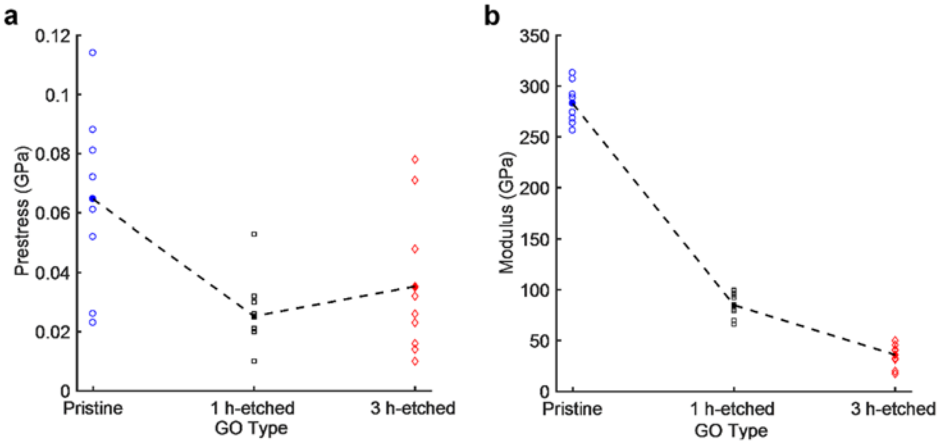

Supplementary Figure 4. Summary statistics for elastic modulus and pre-stress in pristine and etched GO. (a) Summary statistics plot for the elastic modulus as a function of GO etching time. (b) Summary statistics for pre-stress in GO as a function of etching time. In (a) and (b), hollow symbols represent experimental data points while solid symbols represent their average values. A dashed line is used to connect the average values in (a) and (b) to show an overall decrease in properties with increasing etching time.

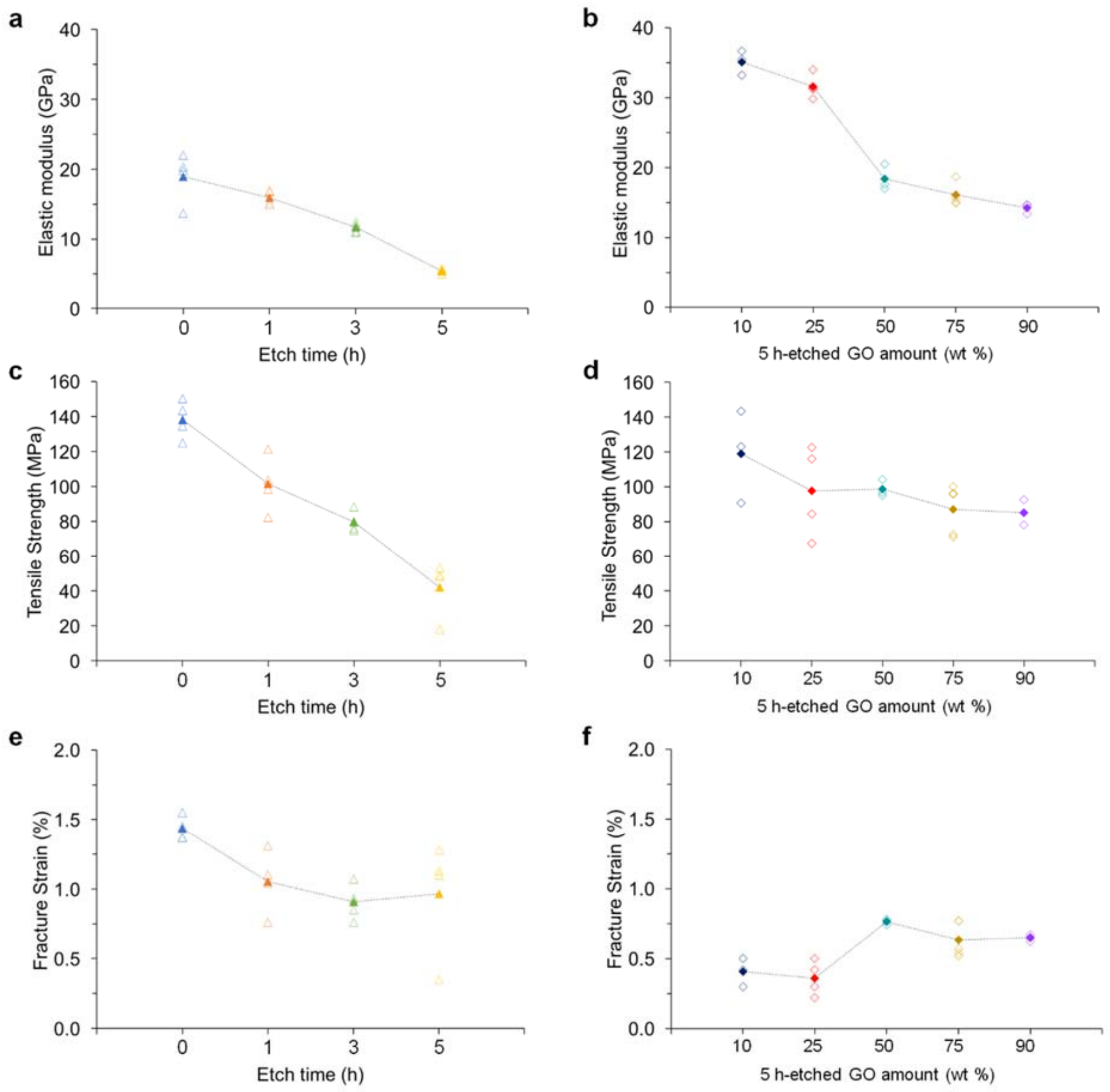

Supplementary Figure 5. Summary statistics for elastic modulus, tensile strength, and fracture strain of non-mixed and mixed GO multilayer films. Plots of the (a, b) elastic moduli, (c, d) tensile strengths, and (e, f) fracture strains of non-mixed and mixed films as a function of GO etching time (a, c, e) or film composition (b, d, f). Compared to non-mixed films, mixed films maintain a greater percentage of their original stiffness and strength as the overall porosity of the constituents increases. In (b), (d), and (f), the notation x wt % indicates a composition of x wt % 5 h-etched GO and $(100 - x)$ wt % pristine GO. Hollow symbols represent experimental data points while solid symbols represent average values. A dashed line is used to connect average values to show overall trends.

Supplementary Tables

Supplementary Table 1. Elastic modulus and pre-stress obtained in our nanoscale membrane deflection experiments based on linear-elastic analysis from force-deflection curves.

System	Modulus (GPa)	Pre-stress (GPa)
GO	283 ± 21	0.07 ± 0.03
1 h-etched GO	85 ± 12	0.03 ± 0.01
3 h-etched GO	36 ± 11	0.04 ± 0.02

Supplementary Table 2. Measured elastic modulus and tensile strength of pristine, etched, and mixed GO multilayer films. The notation x wt % indicates a composition of x wt % 5 h-etched GO and $(100 - x)$ wt % pristine GO.

Entry	System	Elastic modulus (GPa)	Tensile strength (MPa)	Fracture strain (%)
1	GO	19.0 ± 3.6	138 ± 11	1.4 ± 0.1
2	1 h-etched GO	16.6 ± 1.7	90 ± 29	1.0 ± 0.4
3	3 h-etched GO	11.7 ± 0.7	80 ± 5	0.9 ± 0.1
4	5 h-etched GO	5.4 ± 0.3	42 ± 16	1.0 ± 0.4
5	10 wt %	35.1 ± 1.7	119 ± 27	0.4 ± 0.1
6	25 wt %	31.6 ± 1.8	98 ± 26	0.4 ± 0.1
7	50 wt %	18.4 ± 2.8	97 ± 11	0.7 ± 0.2
8	75 wt %	16.1 ± 1.5	87 ± 14	0.6 ± 0.1
9	90 wt %	14.2 ± 0.8	85 ± 7	0.7 ± 0.0

Supplementary Methods

Materials and instrumentation. Unless otherwise stated, all reagents were used as received. Graphite powder (grade 2012) was purchased from Asbury Carbons (Asbury, NJ). Potassium permanganate was purchased from Sigma-Aldrich Co., LLC (Milwaukee, WI). Concentrated sulfuric acid (H_2SO_4) and hydrochloric acid (HCl; 37 wt % in water) were purchased from Junsei Chemical Co., Ltd. (Japan). Hydrogen peroxide (H_2O_2; 30 wt % in water) and ammonium hydroxide solution (NH_4OH; 30% NH_3 basis) were purchased from Sigma-Aldrich Co., LLC (Milwaukee, WI) and refrigerated during storage. Ultrapure deionized water (resistivity 18.2 MΩ cm) was obtained from a Direct Q3 system (Millipore Inc., Billerica, MA). Silicon wafers (Item #785, 100 mm diameter, p-type, B-doped, single-side-polished) were purchased from University Wafer, Inc. (Boston, MA).

X-ray photoelectron spectroscopy (XPS) experiments were carried out on a Thermo Scientific Theta Probe ARXPS (Al Kα radiation, hv = 1486.6 eV; Thermo Fisher Scientific Inc., West Palm Beach, FL) equipped with an electron flood gun. Pristine and etched graphene oxide (GO) films for XPS analysis were prepared by vacuum-assisted filtration of pristine and etched GO solution, respectively, through a hydrophilic polyvinylidene fluoride (PVDF) membrane (HVLP04700, 0.45 μm pore size, Millipore Inc., Billerica, MA). Raman spectroscopy measurements were collected on a NRS-3100 Raman spectrometer (Jasco Inc., Easton, MD) with a 514 nm laser excitation. High-resolution transmission electron microscopy (HR-TEM) images were taken using an aberration-corrected FEI Titan 80-300 (FEI Co., Hillsboro, OR) at an accelerating voltage of 80 kV. The spherical aberration was set to ca. −2.231 μm and images were taken at a defocus value of ca. +10 nm. Scanning electron microscopy (SEM) images of supported nanosheets were taken using a Hitachi S-4800 microscope (Hitachi High-Tech. Co., Japan). SEM images of multilayer films were obtained using a Hitachi SU8030 microscope (Hitachi High-Tech. Co., Japan). Atomic force microscopy (AFM) images of suspended nanosheets were obtained in tapping mode using a Dimension 3100 AFM system (Veeco Instruments Inc., Plainview, NY), respectively. Water-contact angles of the silicon substrates were measured using a VCA Optima contact angle instrument (AST Products, Inc., Billerica, MA) by dropping 4 μL of ultrapure deionized water onto the substrate, with measurements taken at three different locations on each substrate.

Synthesis of GO. Each batch of graphite oxide was prepared using a modified Hummers method[1]. Briefly, graphite (5 g) and concentrated H_2SO_4 (187 mL) were stirred together and cooled to 0 °C using an ice bath. Potassium permanganate (25 g) was slowly added to this mixture, with the temperature kept below 10 °C. The reaction mixture was then transferred to a 35 °C water bath and stirred for 6 h. Next, the mixture was transferred to an ice bath, and ultrapure deionized water (250 mL) was slowly added, taking care to keep the temperature below 55 °C. Additional ultrapure deionized water (500 mL) was then added, followed by the addition of H_2O_2 (10 mL) until the solution became orange brown. The resulting graphite oxide was filtered and washed with HCl (2 L of a 1 M solution) over a cellulose membrane (Whatman filter paper, 2.5 μm pore size, Millipore Inc., Billerica, MA) overnight. The filtered "cake" of graphite oxide was dispersed in acetone (2 L), then filtered and washed with acetone (4 L) over a cellulose membrane overnight. The resulting filtered "cake" of graphite oxide was then dispersed in ultrapure deionized water with mild sonication for 30 min. Any residual unexfoliated graphite oxide was removed by centrifuging at 1,028 rcf for 30 min using a Gyrozen 1508R centrifuge (Gyrozen, South Korea), with the precipitate discarded. The final dispersions contained ~2 mg mL^{-1} of GO.

Synthesis of porous GO. Porous GO was produced from the as-synthesized GO dispersions. Briefly, NH_4OH (0.5 mL) and H_2O_2 (0.5 mL) were added to the GO dispersion (10 mL). Subsequently, the mixture was placed in an oil bath at 50 °C and kept under magnetic stirring at 30 rpm for a given time (1, 3, or 5 h). After the etching reaction, the excess reactants and by-products in the etched GO solution were separated by centrifuging at 16,421 rcf for 90 min, and removed by discarding the supernatant. The precipitate was diluted with ultrapure deionized water (5 mL) and then further purified by dialysis (Spectra Dialysis Membrane, MWCO: 6,000-8,000, Spectrum Inc., Rancho Dominguez, CA) against an excess amount of ultrapure deionized water (1 L) for at least 3 days. During the dialysis, the ultrapure deionized water was exchanged every 5 h for the first two days, and every 10 h thereafter. The resulting dispersions (~2.8-3.5 mg mL^{-1} depending on the reaction time) were diluted to 2.0 mg mL^{-1} by adding ultrapure deionized water. SEM images of the pristine and etched GO sheets, with the corresponding sheet size distributions (Supplementary Figure 1), show that there are only minor differences in the average sheet size after the etching reaction. The nanopores on pristine and etched GO sheets can be visualized by HR-TEM (Fig. 1). During HR-TEM imaging, we did not observe new pores being generated, or

existing pores being enlarged, suggesting that the pores are neither generated or significantly altered by electron beam irradiation.

Preparation of silicon substrates with microwells. Si substrates patterned with arrays of circular microwells (1.76 µm diameter and 4 µm depth) were fabricated by photolithography and deep reactive-ion etching (DRIE). A 1.2 µm-thick photoresist layer (S1813 positive photoresist manufactured by Dow Electronic Materials Microposit, catalog number DEM-10018348, Capitol Scientific, Inc, Austin, TX) was spin-coated onto the Si wafer at 4000 rpm using a spin coater (Cee 200X, Brewer Science, Inc., Rolla, MO). Following a 1-minute soft bake at 100 °C on a hot plate, the wafer was exposed to UV light (365 nm, 18 mW cm^{-2}) for 4 s on the Mask Aligner instrument (Suss MABA6, SÜSS MicroTec AG, Garching, Germany). After exposure, the wafer was developed in a MF 319 developer (manufactured by Dow Electronic Materials Microposit, catalog number: DEM-10018042, Capitol Scientific, Inc, Austin, TX) for 60 s. Spin-rinsing was carried out with ultrapure deionized water (500 mL) for 30 s at approximately 300 rpm, followed by a 1-minute spin-drying at 3000 rpm.

The resulting photoresist-masked silicon wafer was then subjected to microwell-etching using a DRIE machine (STS LpX Pegasus, SPTS Technologies Ltd, San Jose, CA). After etching, the remaining photoresist was removed using acetone, and the wafer was cleaned using isopropanol and ultrapure deionized water. This wafer was then cleaved into smaller substrates to be used in the Langmuir-Blodgett (LB) deposition and subsequent membrane-deflection experiments.

Prior to LB deposition, the substrates were cleaned using the following procedure: 1) submerged in 2 mL of a 3:1 v/v mixture of conc. H_2SO_4:30 wt % H_2O_2 and heated in an SPX microwave reactor (Biotage AB, Uppsala, Sweden; software version 2.3, build 6250) at 180 °C for 45 min, 2) sonicated for 10 min each in ultrapure deionized water (~10 mL), methanol (~10 mL), and ultrapure deionized water (~10 mL), respectively, 3) dried under a flow of nitrogen for 1 min, and 4) treated with O_2 plasma (5 min at 190 W and 10-15 mTorr O_2) in a Model PC-2000 plasma cleaner (South Bay Technology, Inc., San Clemente, CA). The yield of intact suspended GO membranes is strongly dependent on the water-contact angle of the substrate[7]. Therefore, after this cleaning process, the substrates were left under ambient conditions and their water-contact angle was monitored until the desired values were reached (an optimal water contact angle of ~70°

yielded both intact films and high coverage of GO sheets) prior to LB deposition (see procedure below). The water-contact angle of the freshly plasma-treated substrates was close to 0°, gradually increasing over time, and reaching a maximum of ~95° approximately one week after plasma treatment.

LB assembly of pristine and etched GO single layers. To prepare suspended single-layer pristine and etched GO membranes for the AFM membrane deflection experiments, the LB assembly method was employed[9]. The as-prepared pristine or etched GO dispersion was diluted with MeOH to a mixture of 5:1 v/v MeOH:GO dispersion. The model 116 trough (Nima Technology Ltd., Espoo, Finland) was cleaned with acetone, and filled with ultrapure deionized water. Generally, the pristine or etched GO solution (300-480 μL) was spread onto the water surface dropwise at a rate of 100 $\mu L\ min^{-1}$ using a glass syringe, forming a single-layer film on the surface. A tensiometer attached to a Wilhelmy plate was used to monitor the surface pressure. The film was allowed to equilibrate for at least 20 min after spreading, and then compressed by barriers at a speed of 100 $cm^2\ min^{-1}$. Near the onset of the surface pressure increase, the pristine or etched GO single layer was transferred by vertically dipping the substrate into the trough and slowly pulling it up at a rate of 2 $mm\ min^{-1}$. Substrates with a water contact angle of ~70° were used to ensure deposition of intact suspended membranes.

Atomic force microscopy membrane-deflection tests. A single-crystal diamond probe (catalog number: ART D160, K-TEK Nanotechnology, Wilsonville, OR) was used to indent at the membrane center with an AFM (Dimension 3100, Veeco Instruments Inc., Plainview, NY). The stiffness of the cantilever (k = 3.01 $N\ m^{-1}$) was calibrated using a standard cantilever (CLFC-NOBO, Bruker Co., Billerica, MA)[10]. The tip radius of the AFM probe (R = 15 nm) was measured by an FEI NovaNano 600 SEM. All experiments were carried out at room temperature and 16% humidity inside a customized environmental chamber. A constant deflection rate of 1 $\mu m\ s^{-1}$ was used in all tests. Pre-testing and post-mortem AFM scans were taken for each specimen using tapping AFM imaging, with a tapping amplitude of 1 nm, under frequency control conditions. A scan rate of 0.5 Hz was used for all images in an imaging window of 2 μm.

For a suspended, circular, linear elastic membrane under a central load, the force vs. deflection response can be approximated as[11]

$$F = \pi\sigma_0 h\delta + \frac{Eh}{q^3 a^2}\delta^3 \quad (1)$$

where F is the applied force, δ is the membrane center deflection, h is the effective thickness of the specimen (taken as 0.75 nm)[12], σ_0 is the pre-stress in the membrane, a is the membrane radius, E is the elastic modulus, and q is a dimensionless constant defined as $q(v) = (1.05 - 0.15v - 0.16v^2)^{-1}$ where v is the Poisson's ratio. According to previous density functional-based tight-binding (DFTB) calculations[7,13], the Poisson's ratio of the systems studied here was taken as 0.2. We defined specific guidelines to select the fitting region on the raw data (see Supplementary Note 2) to achieve consistency when fitting the linear elastic behavior of different samples.

Fabrication and mechanical analysis of multilayer films. Pristine and etched GO films were prepared via vacuum-assisted filtration of aqueous dispersions of pristine or etched GO, respectively. Mixed films were similarly fabricated by vacuum-filtering pre-mixed aqueous dispersions containing pristine and 5 h-etched GO sheets in varying weight ratios. All films were prepared from 10 mL of a 2 mg mL^{-1} dispersion, and were filtered over mixed cellulose membranes (MF-Millipore membrane filter, hydrophilic, 0.45 μm pore size, Millipore Inc., Billerica, MA).

Mechanical tensile properties of the multilayer films were evaluated under uniaxial tension using an ElectroForce 5500 mechanical test instrument (TA Instruments, New Castle, DE), equipped with a 50 lbf load cell (TA Instruments Inc., New Castle, DE). Samples for testing were cut from films into rectangular strips (approximately 3 mm wide and 24 mm long) by compression with a razor blade. Sample width and length were measured by digital calipers. Prior to testing, a pre-stress of 0.1 N was applied to all samples to make them taut. A strain rate of 2×10^{-4} min^{-1} was used for testing, and 3-5 strips were analyzed for each film. In all cases, results were obtained from samples that did not break near the clamp. Elastic modulus was calculated from the slope of the linear-elastic portion of the stress-strain curve, and tensile strength was determined as the peak stress obtained. Based on this analysis, the results of the tensile tests are summarized in Supplementary Table 2 and Supplementary Figure 5. After mechanical testing, sample thickness was measured from the fracture cross-section using a FEI NovaNano SEM 600 (FEI Co., Hillsboro, OR). For all of the films, measurements were taken at regular intervals along the length of the entire cross-section, and averaged to yield the film thickness.

Lap-shear tests of the multilayer films were carried out on an Instron 599 tester (Instron Engineering Co., Norwood, MA) equipped with a 2248 lbf load cell (Instron Engineering Co., Norwood, MA). Samples were cut into rectangular strips (4 mm × 3 mm) and fixed between two glass slides (Paul Marienfeld GmbH & Co. KG, Lauda-Königshofen, Germany) using Devcon 2-ton epoxy adhesive (Devcon, Danvers, MA). The samples were first air-dried at room temperature and then placed in a 60 °C oven for 5 h to fully cure the epoxy resin. Two additional pieces of glass were glued to the pulling ends of the slides to compensate for the thickness of the slides (See schematic drawing in Fig. 5a), so that the shear stress is strictly aligned along the in-plane direction of the GO films. For measurement, both ends of the prepared specimen were gripped and pulled in the opposite direction at a strain rate of 0.3 mm min^{-1}. After all the measurements, the fractured surfaces were examined by SEM to ensure the same delaminate from within the GO film, rather than from the interface of GO/adhesive or adhesive glass. Shear stress were calculated by dividing the measured shear force by the area of the GO sample (12 mm^2). Values of shear strength were determined by the stress upon delamination.

Supplementary Notes

Supplementary Note 1. XPS and Raman characterization of pristine and etched GO sheets

The chemical compositions of the pristine and etched nanosheets were monitored by XPS, confirming that the amount of oxidized carbons decreases as etching time is extended. As shown in the C1s XPS scans (Supplementary Figure 2a), the intensity of the peak corresponding to graphitic carbon atoms (284.5 eV) increases with etching time, while that of the peak corresponding to contributions from the oxidized carbon atoms decreases[2-4]. This can be attributed to the generation of nanopores at the oxidized sp^3 domains of GO, which effectively removes oxidized carbons[5]. In addition, the ring-opening of the GO epoxide groups by ammonia during the etching process further contributes to the decrease in oxidized carbons[6]. This reaction effectively removes epoxide groups and produces vicinal amine and hydroxyl species that can react further, leading to reduction of the carbon backbone[7]. The reduction of GO during the etching process is further supported by the Raman spectroscopy results, which show that the ratio of the intensity of D and G peaks (I_D/I_G) gradually decreases with etching time (Supplementary Figure 2b). This observation suggests that nanopores are generated through the sacrifice of the oxidized sp^3 domains of GO[8].

Supplementary Note 2. Analysis of membrane-deflection tests

The analysis of AFM force-deflection curves for GO single layers with different porosity necessitates establishing a consistent set of criteria to determine the region of the curve which will be fit using the linear elastic force-deflection model[11]. The selection of the first point of the fitted region, which corresponds to the point of contact in which the membrane has undergone no applied load, is selected to be the point at which the force in the AFM cantilever matches the average force measured as the tip approaches the membrane, as shown in Supplementary Figure 3a. By selecting this point, adhesive effects are eliminated given that the cantilever has straightened after snapping into the membrane, thereby making the effective force in the cantilever zero. The final point of the fitted region must correspond with the first deviation of linear elasticity during membrane deflection. This selection can be guided by manipulating the linear elastic, force-deflection model into the form

$$\ln F \approx 3\ln\delta + \ln\left(\frac{Eh}{q^3 a^2}\right) \tag{2}$$

which is valid only for large membrane deflections (Supplementary Figure 3b). Here, F is the applied force, δ is the deflection at the center of the membrane, E is the elastic modulus of the membrane, h is the membrane thickness (taken as 0.75 nm)[12], a is the membrane radius (here, a = 1760 nm according to SEM and AFM characterization) and q is a dimensionless parameter, given by $q(v) = (1.05 - 0.15v - 0.16v^2)^{-1}$. In this last expression, v is the Poisson's ratio of the membrane, taken to be 0.2 according to previous literature[7]. After selection of the two fit extremum, experimentally obtained force-deflection curves are fit (Supplementary Figure 3c) to the linear-elastic model with an in-house MATLAB code (Version 2012a, MathWorks).

A summary of the results obtained by fitting the force-deflection curves is shown in Supplementary Table 1 and Supplementary Figure 4. As expected, the elastic modulus of the material is strongly dependent on the total porosity of the sample, while pre-stress is seemingly independent of etching time. In addition, and in agreement with the stress concentration behavior of defects, the average rupture force decreases monotonically with increasing etching time, as shown in Supplementary Figure 3c. Notably, the 5 h-etched GO single layers were found to rupture during LB deposition due to the substantial pore size in the membranes, which led to significant stress concentration and weakening of the material. While a low number of intact membranes were found, these did not

exhibit mechanical properties consistent with 5 h-etched GO single layers (E = 117 ± 23 GPa and σ_0 = 0.05 ± 0.03 GPa), and these results were not included in our mechanical analysis.

Supplementary References

1 Kim, F. *et al.* Self-propagating domino-like reactions in oxidized graphite. *Adv. Funct. Mater.* **20**, 2867-2873 (2010).

2 Yang, D. *et al.* Chemical analysis of graphene oxide films after heat and chemical treatments by X-ray photoelectron and micro-Raman spectroscopy. *Carbon* **47**, 145-152 (2009).

3 Ganguly, A., Sharma, S., Papakonstantinou, P. & Hamilton, J. Probing the thermal deoxygenation of graphene oxide using high-resolution in situ X-ray-based spectroscopies. *J. Phys. Chem. C* **115**, 17009-17019 (2011).

4 Koinuma, M. *et al.* Analysis of reduced graphene oxides by X-ray photoelectron spectroscopy and electrochemical capacitance. *Chem. Lett.* **42**, 924-926 (2013).

5 Han, T. H., Huang, Y.-K., Tan, A. T. L., Dravid, V. P. & Huang, J. Steam etched porous graphene oxide network for chemical sensing. *J. Am. Chem. Soc.* **133**, 15264-15267 (2011).

6 Compton, O. C., Dikin, D. A., Putz, K. W., Brinson, L. C. & Nguyen, S. T. Electrically conductive "alkylated" graphene paper via chemical reduction of amine-functionalized graphene oxide paper. *Adv. Mater.* **22**, 892-896 (2010).

7 Wei, X. *et al.* Plasticity and ductility in graphene oxide through a mechanochemically induced damage tolerance mechanism. *Nat. Commun.* **6**, 8029 (2015).

8 Park, H. *et al.* Large scale synthesis and light emitting fibers of tailor-made graphene quantum dots. *Sci. Rep.* **5**, 14163 (2015).

9 Cote, L. J., Kim, F. & Huang, J. Langmuir−Blodgett assembly of graphite oxide single layers. *J. Am. Chem. Soc.* **131**, 1043-1049 (2009).

10 Tortonese, M. & Kirk, M. Characterization of application specific probes for SPMs. *Proc. Soc. Photo. Opt. Instrum. Eng.* **3009**, 53-60 (1997).

11 Lee, C., Wei, X., Kysar, J. W. & Hone, J. Measurement of the elastic properties and intrinsic strength of monolayer graphene. *Science* **321**, 385-388 (2008).

12 Rezania, B., Severin, N., Talyzin, A. V. & Rabe, J. P. Hydration of bilayered graphene oxide. *Nano Lett.* **14**, 3993-3998 (2014).

13 Soler-Crespo, R. A. *et al.* Engineering the mechanical properties of monolayer graphene oxide at the atomic level. *J. Phys. Chem. Lett.* **7**, 2702-2707 (2016).
